# Fabrication of Low-Temperature ppb-Level Ethanol Gas Sensor Based on Hierarchical NiO-SnO_2_ Nanoflowers Under Hydrothermal Conditions

**DOI:** 10.3390/nano15191471

**Published:** 2025-09-25

**Authors:** Liming Song, Xiaoxin Dou, Jianmei Shao, Yuanzheng Luo, Fumiao Liu, Chengyong Li, Lijuan Yan, Chuhong Wang, Yuting Li, Yuqing Cai, Jinsheng He, Zhenqing Dai, Ruikun Sun, Qin Xie

**Affiliations:** 1 School of Electronics and Information Engineering, Guangdong Ocean University, Zhanjiang 524088, China; songlm@gdou.edu.cn (L.S.); douxx738@stu.gdou.edu.cn (X.D.); shaojm89@163.com (J.S.); luoyz@gdou.edu.cn (Y.L.); 17664067713@stu.gdou.edu.cn (F.L.); ylj_gdou@126.com (L.Y.); wchong@gdou.edu.cn (C.W.); liyuting5892022@163.com (Y.L.); caiyuqing521825@163.com (Y.C.); 11911405rr@stu.gdou.edu.cn (J.H.); 2 Analyzing and Testing Center, Guandong Ocean University, Zhanjiang 524088, China; daizq@gdou.edu.cn (Z.D.); sunrk@gdou.edu.cn (R.S.)

**Keywords:** NiO-SnO_2_, gas sensor, ethanol, p-n heterojunction, nanoflowers

## Abstract

Hierarchical NiO-SnO_2_ nanoflowers were prepared via a one-step hydrothermal method. The morphology, structure and components of the hierarchical NiO-SnO_2_ nanoflowers were examined via scanning electron microscopy (SEM), transmission electron microscopy (TEM), X-ray powder diffraction (XRD) and X-ray photoelectron spectroscopy (XPS), respectively. The ethanol gas-sensing performance was systematically analyzed between pure hierarchical SnO_2_ nanoflowers and the hierarchical NiO-SnO_2_ nanoflowers. The results indicated that the hierarchical NiO-SnO_2_ nanoflowers showed better gas-sensing properties than the pure hierarchical SnO_2_ nanoflowers at 164 °C. The enhanced gas-sensing performance was ascribed to the formation of p-n heterojunctions between p-type NiO and n-type SnO_2_. Additionally, NiO has a catalytic role. Therefore, hierarchical NiO-SnO_2_ nanoflowers could be a potential gas-sensing material for the fabrication of high-quality ethanol gas sensors.

## 1. Introduction

Nowadays, with developments in science and technology, atmospheric pollution is becoming an extensive and highly concerning problem all over the world. In order to resolve this problem, gas sensors based on metal oxide semiconductors have attracted widespread attention in applications such as human health [1,2], environmental safety [3,4], gas leakage [5,6] and air quality detection [7,8] owing to their ease of fabrication, low cost and energy consumption and excellent gas-sensing performance regarding various harmful gases. Among them, tin oxide (SnO_2_), an important wide-band-gap n-type semiconductor (Eg = 3.6 eV) with good chemical and physical properties [9,10]. It has been extensively applied in many areas, including photocatalysts [11,12], dye-sensitized solar cells [13,14], quantum dots [15,16], lithium-ion batteries [17,18] and gas sensors [19,20]. However, gas sensors based on pure SnO_2_ sensing materials usually work at extremely high temperatures and show low gas sensitivity [21,22], which limits the application of this type of gas sensor. Therefore, an increasing number of researchers are seeking methods to improve the gas-sensing performance. For example, they have designed hierarchical structures [23,24], doped noble and rare earth metals [25,26], synthesized porous structures [27,28], fabricated composite heterojunctions [29,30] and so on. Owing to the synergistic effects of the composite configuration, it has been found that the fabrication of composite heterojunction materials renders the sensor highly sensitive and selective [31,32]. For example, Shi et al. fabricated a p-n heterojunction consisting of two-dimensional MoS_2_ nanoflakes vertically grown on one-dimensional SnO_2_ nanotubes via electrospinning and a subsequent hydrothermal route. The results indicated that the MoS_2_/SnO_2_ composites showed high sensitivity to NO_2_ compared to pure SnO_2_ nanotubes at room temperature [33]. Qin et al. synthesized SnO_2_/Co_3_O_4_ nanocomposites via a straightforward pyrolytic bimetallic organic framework method. A heterojunction was obtained via a simple hydrothermal method. The formed SnO_2_/Co_3_O_4_ nanocomposite sensor exhibited greater sensitivity and selectivity to H_2_ than pure SnO_2_ nanoparticles in mixed gas environments at 325 °C [34]. Lu et al. prepared SnO_2_-CuO core–shell nanowires with a precisely controlled shell thickness through a sequential process combining a solution process and atomic layer deposition. The sensor based on SnO_2_-CuO core–shell nanowires presented higher gas sensitivity to formaldehyde than pure SnO_2_ at 250 °C [35]. Although these works have effectively improved the gas-sensing performance of pure SnO_2_ nanomaterials, SnO_2_ gas sensors still require more accurate responses to target gases, and their selectivity in identifying target gases in the presence of other gases at low working temperatures must be enhanced. Therefore, it is extremely important to find a suitable additive to construct a heterostructure with SnO_2_.

Nickel oxide (NiO) is an important p-type semiconductor for fabricating functional layers with unique properties such as high chemical and thermal stabilities [36,37]. Those properties make the NiO layer an appropriate material for various applications, including a catalyst [38,39], fuel cell electrode [40,41], electrochemical supercapacitor [42,43], magnetic material [44,45], gas sensor [46,47] and so on. Currently, researchers focused on p-type MOS gas sensors are heavily outnumbered by those working on n-type oxide semiconductors. This is because the carrier mobility of p-type oxide semiconductors is comparatively lower than that of n-type oxide semiconductors [48,49], leading to inferior gas-sensing performance. For example, Zhang et al. obtained NiO core-like nanochains by calcining Ni_2_C_2_O_4_ nanorods and employed them as methane sensing materials. The results show that the sensor based on NiO coral-like nanochains exhibited 3.6% gas sensitivity towards 100 ppm CH_4_ at an operating temperature of 320 °C [50]. Nakate et al. prepared two-dimensional nanocrystalline NiO nanoparticles using a chemical route. The results indicate that the response of the NiO nanoparticle sensor to 100 ppm acetaldehyde is 108% at 250 °C [51]. Singh et al. synthesized nanocrystalline NiO thin films via RF magnetron sputtering. And the results show that the NiO thin film sensor displayed the highest response of 28.8 for 200 ppm H_2_S at 400 °C [52]. Although both experiments and theory indicate that NiO gas sensors suffer from low sensitivity and high working temperature, which limits their practical application, NiO has advantages of volatile organic compound (VOC) catalytic activity and oxygen adsorption characteristic, which makes it can be a suitable addictive to fabricate composites with other metal oxide semiconductor sensing materials and enhance gas sensing performance.

In this study, the hierarchical NiO-SnO_2_ nanoflowers heterojunction has been fabricated through the hydrothermal method and employed as an ethanol gas sensor. The intimate p-n junction formed at the NiO-SnO_2_ interface effectively modulates interfacial charge transport, leading to markedly enhanced sensing performance. The gas sensing examinations clearly indicate that the hierarchical NiO-SnO_2_ nanoflowers heterojunction displayed better gas sensing properties towards ethanol compared to pure hierarchical SnO_2_ nanoflowers, including short response and recovery time and high selectivity at 164 °C. Notably, the limit of detection (LOD) to ethanol of the obtained hierarchical NiO-SnO_2_ nanoflowers heterojunction gas sensor reaches 500 ppb, representing a substantial improvement in the sensing capability of the gas sensor based on SnO_2_ materials.

## 2. Materials and Methods

### 2.1. Preparation of Hierarchical NiO-SnO_2_ Nanoflowers

The pure hierarchical SnO_2_ nanoflowers were prepared in our previous work [53]. Based on this work, the hierarchical NiO-SnO_2_ nanoflowers heterojunction was prepared by a hydrothermal method after calcination. Initially, 2.2167 g of stannous chloride dihydrate and 0.0286 g of nickel nitrate hexahydrate were successively dissolved in 35 mL of deionized water under vigorous stirring for 1 h to yield a homogeneous solution. Subsequently, 1.6034 g sodium hydroxide and 0.7288 g CTAB were added to the above solution, respectively. Then the mixed solution was transferred into a 50 mL Teflon-lined stainless autoclave after stirring for 5 h, and kept at 180 °C for five hours in a furnace. After the autoclave was cooled down naturally, the product was collected by centrifugation and washed with distilled water several times before drying an oven at 70 °C for 1 h. Finally, the as-obtained products were annealed in a muffle furnace at 400 °C for 3 h to obtain 0.45% NiO-doped SnO_2_ nanoflowers. All the above reagents employed in the preparation of hierarchical NiO-SnO_2_ nanoflowers were analytical reagent grade and used without further purification. Stannous chloride dihydrate (SnCl_2_·2H_2_O, 99.9%, Sigma-Aldrich, Shanghai, China), Nickel nitrate hexahydrate (Ni (NO_3_)_2_·6H_2_O, 99.99%, Aladdin, Shanghai, China), Metyltrimethyl ammonium bromide (CTAB, 99.9%, Sigma-Aldrich, Shanghai, China), Sodium hydroxide (NaOH, 99.9%, Sigma-Aldrich, Shanghai, China).

### 2.2. Characterizations

The crystalline and phase structure of the samples was characterized by X-ray diffraction (XRD, ADX-2700D X-ray, MA, USA), ADX-2700D X-ray Powder Diffraction Instrument with Cu_Kα_ radiation (λ = 1.15406 Å) in the range of 5° and 90°. The chemical composition and surface properties of the products were investigated by X-ray photoelectron spectra (XPS, Thermo Fisher ESCALAB 250Xi, EG, UK). The morphology and microstructure of the samples were observed by scanning electron microscopy (SEM, Shimadzu SSX-550, Kyoto, Japan) and transmission electron microscopy (TEM, JEOLJEM-200FS, Tokyo, Japan), respectively. The gas-sensing properties of the samples were measured by a CGS-8 intelligent gas-sensing analysis system (Beijing Elite Tech Co., Ltd., Beijing, China). The gas sensing analysis system was evaluated under a static gas distribution method. Firstly, aged gas sensors were inserted into the test sockets and covered with a closed organic glass mask. Then, the test instrument with ten sensor sockets was turned on to record the ambient temperature and humidity, while the operating currents were adjusted to regulate the heating temperature. Subsequently, a specific volume of liquid target gas was drawn up with a syringe, and the gaseous target gas was injected into the 1 L glass bottle. The sensor was removed from the glass bottle when the resistance of the sensor remained stable. Finally, the resistance variation in the sensor in the presence of the target gas can be continuously monitored, and the response of the gas sensor for the target gas was calculated.

### 2.3. Fabrication and Measurement of Gas Sensor

**Figure 1:**
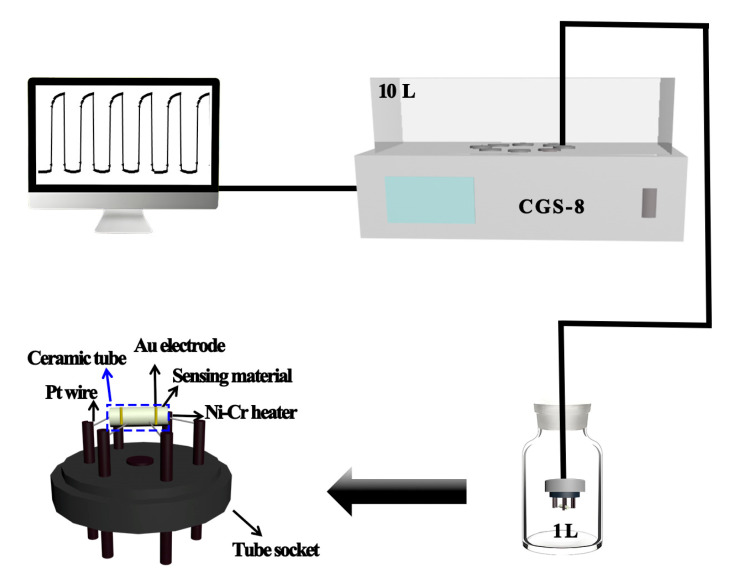
The gas testing system and schematic structure of the hierarchical NiO-SnO_2_ gas sensor.

The fabrication of the gas sensor is described as follows: the as-prepared hierarchical NiO-SnO_2_ nanoflowers and pure hierarchical SnO_2_ nanoflowers were dissolved with a little deionized water to form a paste, respectively. After that, the as-obtained pastes were coated uniformly onto an alumina ceramic tube with a pair of gold electrodes and four Pt wires. Then, a Ni-Cr alloy wire coil that was utilized as a heater was inserted into a ceramic tube to control the operating temperature by adjusting the electrical current. Finally, the tube was welded to the sensor pedestal. The response of the sensor is defined as the ratio of the resistance in air (Ra) and the resistance in target gas (Rg) [54,55]. The gas testing system and schematic structure of the hierarchical NiO-SnO_2_ nanoflowers gas sensor are shown in Figure 1. All gas sensing measurements of hierarchical NiO-SnO_2_ nanoflowers gas sensor were carried out under an ambient relative humidity of 45%.

## 3. Results and Discussion

**Figure 2:**
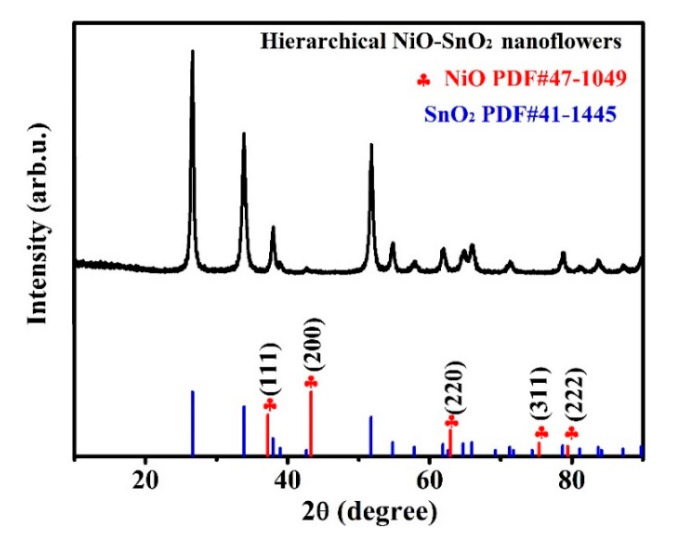
XRD patterns of as-prepared hierarchical NiO-SnO_2_ nanoflowers.

The phase and crystal structure of hierarchical NiO-SnO_2_ nanoflowers were confirmed by XRD measurement, as shown in Figure 2. From this picture, it can be clearly seen that most of the diffraction peaks can be well assigned to the SnO_2_ and NiO, which demonstrates that the as-obtained product was made up of SnO_2_ and NiO. And all diffraction peaks of NiO and SnO_2_ were in good agreement with the standard SnO_2_ (JCPDS: No. 41-1445) and NiO (JCPDS: No. 47-1049) card without any other impurity peaks in this composite [56]. Additionally, the diffraction peaks of hierarchical NiO-SnO_2_ are sharp and strong, which indicates that the as-obtained products have high purity and good crystallinity.

**Figure 3:**
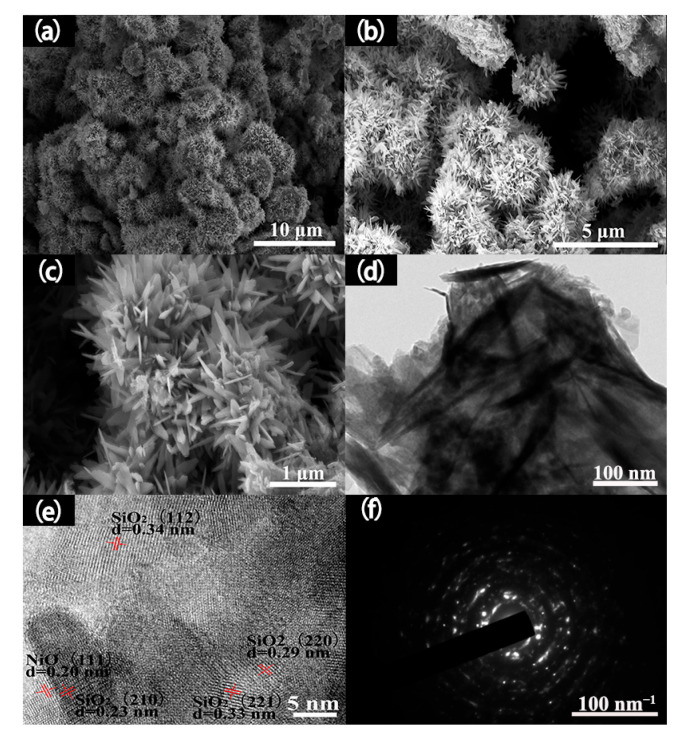
(**a**–**c**) SEM images; (**d**) TEM image; (**e**) HRTEM image, and (**f**) the corresponding SAED pattern of the as-synthesized hierarchical NiO-SnO_2_ nanoflowers.

Figure 3a–c show the SEM images of the hierarchical NiO/SnO_2_ nanoflowers. The micrographs reveal that the as-prepared NiO/SnO_2_ nanocomposite possesses a well-defined hierarchical flower-like architecture and exhibits excellent morphological uniformity. To further elucidate the microscopic structure of the hierarchical NiO/SnO_2_ nanoflowers, TEM measurements were performed, as presented in Figure 3d–f. Figure 3e,f indicate that the crystallites within the sample are polycrystalline, highly crystalline, and essentially free of observable defects. The lattice fringes of 0.20 nm, 0.23 nm, 0.33 nm, 0.29 nm, and 0.34 nm in Figure 3e correspond to the interplanar spacings of the (111) plane of NiO and the (210), (221), (220), and (112) planes of SnO_2_, respectively. Notably, adjacent n-type SnO_2_ crystallites are in intimate contact with p-type NiO crystallites, thereby forming p–n heterojunctions at the interfaces and significantly enhancing the gas-sensing performance of the material.

**Figure 4:**
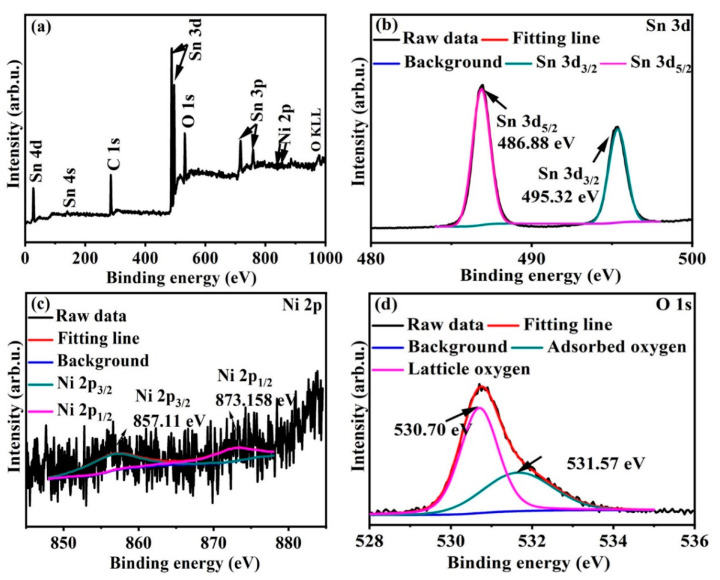
X-ray photoelectron spectroscopy measurement of hierarchical NiO-SnO_2_ nanoflowers. (**a**) The full rang XPS spectrum; (**b**) Sn 3d; (**c**) Ni 2p; (**d**) O 1s.

The XPS measurement was performed to further investigate the surface compositions and chemical state of hierarchical NiO-SnO_2_ nanoflowers. The full range spectrum of the as-obtained product was presented in Figure 4a. From Figure 4a, it is clearly seen that the hierarchical NiO-SnO_2_ nanoflowers were composed of Sn, O, Ni. And the high-resolution XPS spectra for Sn 3d and Ni 2p were shown in Figure 4b,c. As shown in Figure 4b, the peaks located at 495.32 eV and 486.88 eV are ascribed to the Sn 3d_3/2_ and Sn 3d_5/2_, respectively, which indicates that the Sn element exists as Sn^4+^ in the as-obtained sample. From Figure 4c, the peaks centered at 837.16 eV and 857.11 eV are attributed to the Ni 2p_1/2_ and Ni 2p_3/2_, respectively, which demonstrates the existence of Ni in the hierarchical nanoflowers. Additionally, the spectrum of O 1s can be deconvoluted into two peaks centered at 530.70 eV and 531.57 eV, which are ascribed to the lattice oxygen and adsorbed oxygen, respectively. Based on these results, it is very clearly indicated that the as-obtained product is composed of NiO and SnO_2_ nanomaterials.

## 4. Gas Sensing Characteristics

**Figure 5:**
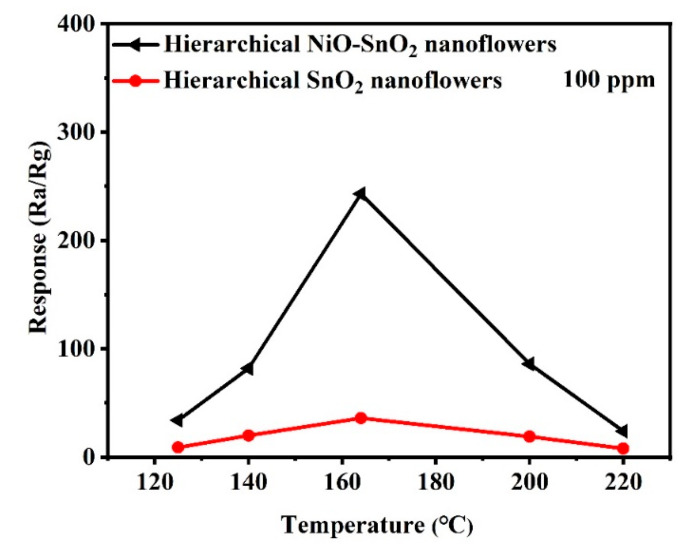
Gas response of the sensor based on hierarchical SnO_2_ nanoflowers and hierarchical NiO-SnO_2_ nanoflowers to 100 ppm ethanol gas at different operating temperatures.

To investigate the effect of NiO doping on hierarchical SnO_2_ nanoflowers gas sensing performance, pure hierarchical SnO_2_ nanoflowers and hierarchical NiO-SnO_2_ nanoflowers gas sensors were designed, and the gas sensing properties to ethanol were examined. It is well known that the operating temperature has an important influence on the gas sensing behavior of the sensors. And the relationship between the gas response and the operating temperature has been studied to explore the optimal operating temperature, as displayed in Figure 5. As shown in Figure 5, it is obvious that the responses to ethanol for both sensors increase firstly and then decrease when the operating temperature is increased. This phenomenon is attributed to the kinetics and thermodynamics of gas adsorption and desorption on the sensing material surface [57]. The rate of reaction between ethanol and surface-adsorbed oxygen species will be increased when the operating temperature is increased. But when the operating temperature is more than the optimal operating temperature, the response of the sensor will show a decrease tendency, which is attributed to the ethanol adsorption active site decreasing caused by the desorption rate is faster than the adsorption rate. Meanwhile, the catalytic role of NiO also promotes the hierarchical NiO-SnO_2_ nanoflower heterojunction gas sensor owns good gas sensing response and low working temperature [58]. Therefore, the optimal operating temperature for both sensors based on the hierarchical pure SnO_2_ nanoflowers and hierarchical NiO-SnO_2_ nanoflowers is determined to be 164 °C.

**Figure 6:**
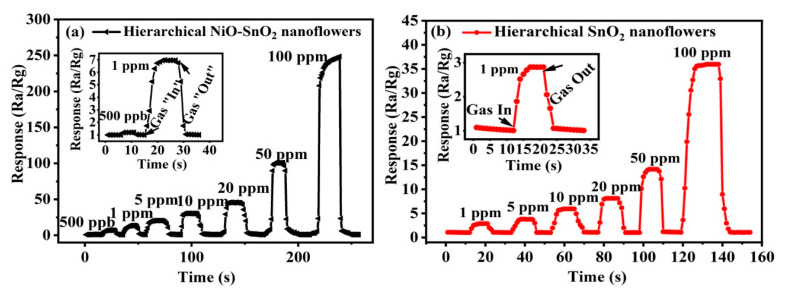
Dynamic gas sensing response and recovery curve of the sensor based on the hierarchical SnO_2_ nanoflowers and hierarchical NiO-SnO_2_ nanoflowers sensors to 1 ppm–50 ppm (**a**) and 500 ppb-50 ppm (**b**), respectively.

In order to deeply investigate the gas sensing characteristic of the sensor, the response and recovery performance of hierarchical SnO_2_ nanoflowers and hierarchical NiO-SnO_2_ nanoflowers were comparatively measured in the ethanol gas ranging from 1 ppm to 50 ppm and 500 ppb to 50 ppm at 164 °C, as shown in Figure 6. From this picture, it can be clearly observed that the gas sensor based on hierarchical SnO_2_ nanoflowers and hierarchical NiO-SnO_2_ nanoflowers both showed good gas sensing properties towards ethanol gas. Their response increases rapidly and then returns to the original value after the ethanol is removed. The hierarchical NiO-SnO_2_ nanoflowers gas sensor shows much better gas sensitivity than the hierarchical SnO_2_ nanoflowers gas sensor. Moreover, the hierarchical NiO-SnO_2_ nanoflowers have a 500 ppb (1 ppm) low detection limit at 164 °C, and the response value is 1.2 (2.86). And the response and recovery times are 4 s (3 s) and 6 s (4 s). The above results demonstrate that the as-prepared hierarchical NiO-SnO_2_ nanoflowers gas sensor owns outstanding gas sensing properties and will be a good candidate for an ethanol gas sensor. And in Table 1, several reported SnO_2_-based ethanol gas sensors are listed for comparison. Compared to other SnO_2_-based ethanol gas sensors, the hierarchical NiO-SnO_2_ nanoflowers gas sensor in our work exhibits better gas sensing performance for ethanol gas molecules at a relative low working temperature.

**Figure 7:**
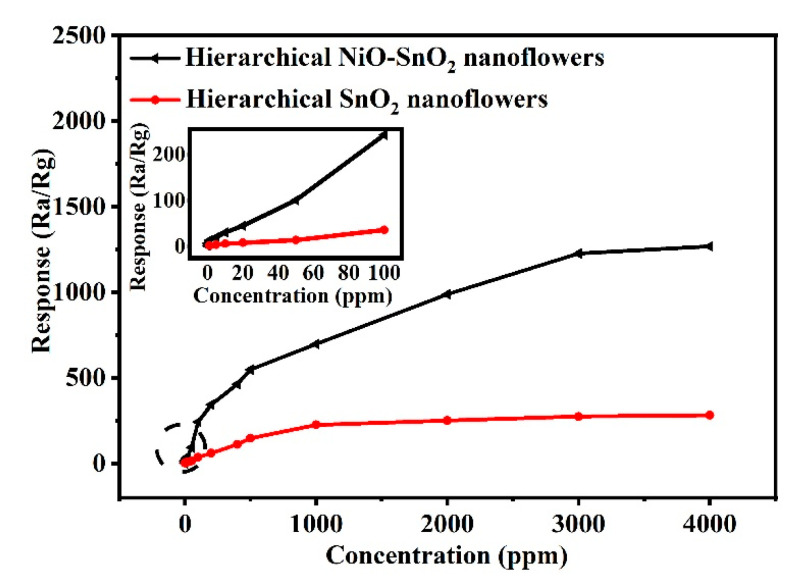
The responses of sensors based on hierarchical SnO_2_ nanoflowers and hierarchical NiO-SnO_2_ nanoflowers towards different concentrations of ethanol gas at 164 °C.

Figure 7 presents the dynamic response curves of the hierarchical SnO_2_ nanoflower sensor and the hierarchical NiO-SnO_2_ nanoflower sensor to ethanol at 164 °C. The responses of both hierarchical SnO_2_ nannoflowers and hierarchical NiO-SnO_2_ nanoflower sensors rise sharply with increasing ethanol concentration, but the increase slows markedly once the concentration of ethanol is more than a fix value and, finally, the response of two sensors increased very slow and then keep stable, which could be explained by the theory that surface adsorptions gradually saturated with the increasing concentration [63,64]. More importantly, the response of the hierarchical NiO-SnO_2_ nanoflowers sensor exhibits a higher response value than that of the hierarchical SnO_2_ nanoflowers sensor towards different ethanol concentrations ranging from 1 ppm to 50 ppm. The hierarchical NiO-SnO_2_ nanoflowers sensor can also detect ethanol gas with a response of 1.2 when the concentration of ethanol is 500 ppb. The above results demonstrate that NiO doped into SnO_2_ can efficiently improve the gas sensing properties, and this hierarchical NiO-SnO_2_ nanoflowers gas sensor is suitable for the detection of ethanol at a low concentration.

**Figure 8:**
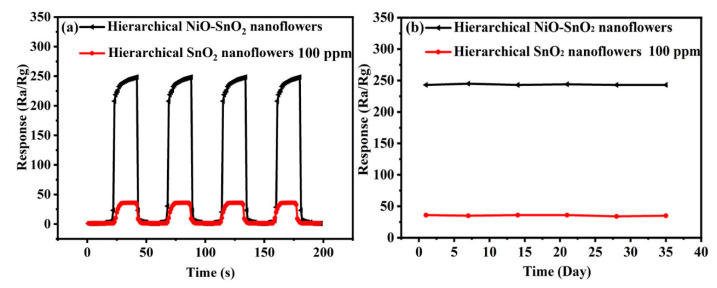
(**a**) Reproducibility and (**b**) stability of the sensor based on hierarchical SnO_2_ nanoflowers and hierarchical NiO-SnO_2_ nanoflowers to 100 ppm ethanol at 164 °C.

The reproducibility and stability are important index to evaluate gas sensing properties of the as-obtained gas sensors [65]. Figure 8a displayed the response and recovery curves with four cycles of hierarchical SnO_2_ nanoflowers and hierarchical NiO-SnO_2_ nanoflowers. As shown in this picture, there is no clear change in the response value after four cycles of gas sensing examination to 100 ppm ethanol at 164 °C, which illustrates that the hierarchical SnO_2_ nanoflowers and hierarchical NiO-SnO_2_ nanoflowers sensors have reproducibility. Meanwhile, the stability of the two sensors has been measured in Figure 8b. From this picture, it can be clearly seen that the sensors almost show a similar response value after 35 days, which demonstrates that the sensors made of hierarchical SnO_2_ nanoflowers and hierarchical NiO-SnO_2_ nanoflowers sensors exhibit long-term stability.

**Figure 9:**
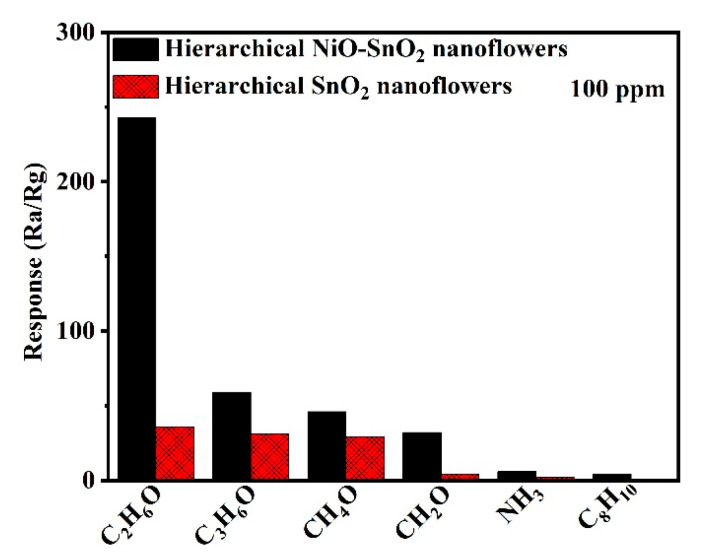
Response of hierarchical NiO-SnO_2_ nanoflowers and hierarchical SnO_2_ nanoflowers towards 100 ppm ethanol, acetone, methanol, formaldehyde, ammonia, and paraxylene gas molecules at 164 °C.

The sensor can display different adsorption and catalytic performance to different kinds of gases, which makes it show different response values towards them and detects a specific gas among them [66]. And this kind of ability is defined as selectivity. Figure 9 depicts the response of hierarchical SnO_2_ nanoflowers and hierarchical NiO-SnO_2_ nanoflowers sensors to various kinds of target gases, including 100 ppm ethanol, acetone, methanol, formaldehyde, ammonia, and paraxylene at 164 °C. And the response was 243 (36), 59 (31), 46 (29), 32 (4), 6 (2), and 4 (1), respectively. The sensor possesses different response values to various target gas is ascribed to the different values of the lowest unoccupied molecule orbit energy for the gas, and the surface structure and electronic characteristics have a big effect on its interaction with different kinds of target gas molecules [67]. Clearly, the sensor based on the hierarchical NiO-SnO_2_ nanoflowers exhibited a much greater gas response to target gas as compared to hierarchical SnO_2_ nanoflowers and displayed the highest response and selectivity to ethanol at 164 °C, which demonstrates the gas sensor made of the hierarchical NiO-SnO_2_ nanoflowers can be a good candidate for detecting ethanol among the above target gases and further confirms that the gas sensing performance of hierarchical SnO_2_ nanoflowers has been efficiently improved by doping NiO.

## 5. Gas Sensing Mechanism

It is well known that the gas sensing mechanism for metal oxide semiconductor can be described by the surface-depletion model in which chemisorbed oxygen plays a considerable role. When the hierarchical SnO_2_ nanoflowers sensor is exposed to the ambient air, the oxygen from the air will be absorbed on the surface of hierarchical SnO_2_ nanoflowers and trap electrons from the conduction band of hierarchical SnO_2_ nanoflowers to form absorbed oxygen species [68,69], as illustrated in Equations (1)–(3).

(1) $$O_{2}\left(gas\right)\to O_{2}\left(ads\right)$$

(2) $$O_{2}\left(ads\right)+e^{-}\to O^{2-}\left(ads\right)$$

(3) $$O^{2-}\left(ads\right)+e^{-}\to {2O}^{-}\left(ads\right)$$

Then, a thick electron-depletion layer will form on the surface of hierarchical SnO_2_ nanoflowers, and the resistance of the material will increase. When the sensor is exposed to reductive ethanol, the ethanol molecules will react with the absorbed oxygen species and release the trapped electrons to the conduction band, which leads to the decreased resistance of the sensor. The stable oxygen species on the surface of the sensing material are present when the working temperature of the sensor is less than three hundred degrees [70,71]. The reaction process between oxygen species and ethanol gas can be explained as follows:

(4) $${CH}_{3}{CH}_{2}OH+{6O}^{-}\left(ads\right)\to {2CO}_{2}+{3H}_{2}O+{6e}^{-}$$

However, the gas sensing mechanism of the hierarchical NiO-SnO_2_ nanoflowers is very different of the pure hierarchical SnO_2_ nanoflowers. Obviously, the hierarchical NiO-SnO_2_ nanoflower enhanced the gas sensing performance compared with the pure hierarchical SnO_2_ nanoflowers, which can be ascribed to the formation of a p-n heterojunction structure between the p-type NiO semiconductor and n-type SnO_2_ semiconductor by adding the NiO material. It is well known that SnO_2_ and NiO are a kind of n-type semiconductor with a lot of electrons and a p-type semiconductor with holes, respectively. When the n-type SnO_2_ semiconductor contacts with the p-type NiO semiconductor, the electrons will move from n-type SnO_2_ to p-type NiO, and holes will move from p-type NiO to n-type SnO_2_ to keep the equalization of the Fermi levels, results in the formation of a self-built electric field at the interface region. Therefore, the depletion layer is generated at the heterojunction interface, and a potential barrier at the p-n heterojunction with band bending is also formed [72,73]. When the hierarchical NiO-SnO_2_ nanoflowers were exposed to air, a depletion layer formed on the surface of the hierarchical NiO-SnO_2_ nanoflowers. And the depletion region at the hierarchical NiO-SnO_2_ structure interface will make the resistance of the hierarchical NiO-SnO_2_ nanoflowers in air higher than that hierarchical SnO_2_ nanoflowers. Once the hierarchical NiO-SnO_2_ is exposed to the ethanol gas, the ethanol gas will react with oxygen species and release the electrons to the conduction band and causing the resistance to decrease. In addition, electrons are released from ethanol molecules, and then combine with holes in p-type NiO, leading to a reduction in the concentration gradient on both sides of the p-n heterojunction. Thus, the formation of p-n heterojunction enormously increases the resistance of the hierarchical NiO-SnO_2_ nanoflowers gas sensor in ambient air and decreases the resistance in ethanol molecules, which extremely improves the gas sensing performance of the hierarchical NiO-SnO_2_ nanoflowers sensor.

**Figure 10:**
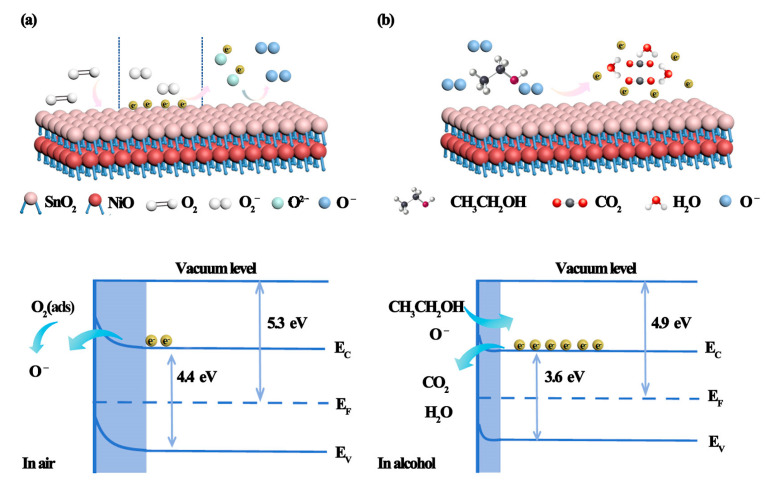
The mechanism diagram (**a**) and energy band structure diagram (**b**) of hierarchical NiO-SnO_2_ nanoflowers for ethanol gas molecules.

In addition, NiO nanomaterials exhibit catalytic activity, significantly enhancing the chemisorptions of oxygen species and the chemical reactions on the NiO-SnO_2_ surface [74,75]. Meanwhile, the hierarchical NiO-SnO_2_ structure is also good for gas diffusion and transport, chemisorptions, and surface reactions. Combining the above results, the hierarchical NiO-SnO_2_ nanoflowers sensor shows outstanding gas sensing properties. The mechanism diagram and energy band structure diagram of hierarchical NiO-SnO_2_ nanoflowers are shown in Figure 10.

## 6. Conclusions

In summary, the hierarchical SnO_2_ nanoflowers and hierarchical NiO-SnO_2_ nanoflowers have been fabricated by a low-cost and environmental friendly hydrothermal method. Compared with pure hierarchical SnO_2_ nanoflowers, the as-obtained hierarchical NiO-SnO_2_ nanoflowers exhibited superior gas sensing behavior and good selectivity toward ethanol at 164 °C. Moreover, the hierarchical NiO-SnO_2_ nanoflowers achieve an ethanol detection limit as low as 500 ppb with a response of 1.2. And the response and recover times are 4 s and 5 s, respectively. The improvement of ethanol response and selectivity may be ascribed to the formation of a better p-n heterojunction between NiO and SnO_2_ and the catalysis of NiO.

## Figures and Tables

**Table 1:** Ethanol gas sensing performance comparison of different SnO_2_-based gas sensors.

Materials	LOD (ppb)	Response Time (s)	Temperature (°C)	Reference
Ag/SnO_2_ composites	2 × 10^5^	80	300	[59]
SnO_2_	1 × 10^5^	3	300	[60]
Pr-doped SnO_2_	2 × 10^3^	12	200	[61]
SnO_2_-ZnO	2 × 10^2^	3	250	[62]
NiO-SnO_2_	5 × 10^2^	1.2	164	This work

## Data Availability

The original contributions presented in this study are included in the article. Further inquiries can be directed to the corresponding author(s).
